# Mapping opportunities and challenges for rewilding in Europe

**DOI:** 10.1111/cobi.12533

**Published:** 2015-05-21

**Authors:** Silvia Ceaușu, Max Hofmann, Laetitia M Navarro, Steve Carver, Peter H Verburg, Henrique M Pereira

**Affiliations:** * German Centre for Integrative Biodiversity Research (iDiv) Halle-Jena-Leipzig, Deutscher Platz 5e04103, Leipzig, Germany; † Institute of Biology, Martin Luther University Halle-WittenbergAm Kirchtor 1, 06108, Halle (Saale), Germany; ‡ Wildland Research Institute, School of Geography, University of LeedsLS2 9JT, United Kingdom; § Institute for Environmental Studies (IVM), VU University AmsterdamDe Boelelaan 1087, 1081, HV, Amsterdam, The Netherlands; ¶ REFER Biodiversity Chair, CIBIO/InBIO, Campus Agrário de VairãoRua Padre Armando Quintas 7, 4485-661, Vairão, Portugal

**Keywords:** biodiversity policy, conservation management, farmland abandonment, land-use change, Natura 2000, rewilding, wilderness, abandono de tierras agrícolas, cambio en el uso de suelo, manejo de la conservación, Natura 2000, naturaleza, políticas de biodiversidad

## Abstract

Farmland abandonment takes place across the world due to socio-economic and ecological drivers. In Europe agricultural and environmental policies aim to prevent abandonment and halt ecological succession. Ecological rewilding has been recently proposed as an alternative strategy. We developed a framework to assess opportunities for rewilding across different dimensions of wilderness in Europe. We mapped artificial light, human accessibility based on transport infrastructure, proportion of harvested primary productivity (i.e., ecosystem productivity appropriated by humans through agriculture or forestry), and deviation from potential natural vegetation in areas projected to be abandoned by 2040. At the continental level, the levels of artificial light were low and the deviation from potential natural vegetation was high in areas of abandonment. The relative importance of wilderness metrics differed regionally and was strongly connected to local environmental and socio-economic contexts. Large areas of projected abandonment were often located in or around Natura 2000 sites. Based on these results, we argue that management should be tailored to restore the aspects of wilderness that are lacking in each region. There are many remaining challenges regarding biodiversity in Europe, but megafauna species are already recovering. To further potentiate large-scale rewilding, Natura 2000 management would need to incorporate rewilding approaches. Our framework can be applied to assessing rewilding opportunities and challenges in other world regions, and our results could guide redirection of subsidies to manage social-ecological systems.

Mapeo de Oportunidades y Retos para el Retorno de la Vida Silvestre

**Resumen:**

El abandono de tierras agrícolas ocurre en todo el mundo debido a factores socio-económicos y ecológicos. En Europa, las políticas ambientales y agrícolas tienen el objetivo de prevenir el abandono y frenar la sucesión ecológica. La reintroducción o el retorno de la vida silvestre (“rewilding”) representa una estrategia alternativa a esto. Desarrollamos un marco de trabajo para evaluar las oportunidades de reintroducción en diferentes dimensiones de naturaleza a lo largo de Europa. Mapeamos la luz artificial, la accesibilidad para humanos con base en la infraestructura de transporte, la proporción de productividad primaria (es decir, la productividad del ecosistema incautado por los humanos por medio de la agricultura o la silvicultura) y la divergencia de vegetación natural potencial en áreas que se proyecta estarán abandonadas para el 2040. A nivel continental, los niveles de luz artificial fueron bajos y la divergencia de vegetación natural potencial fue alta en las áreas de abandono. La importancia relativa de las medidas de naturaleza difirió regionalmente y estuvieron conectadas fuertemente a los contextos ambientales y socio-económicos locales. Las grandes áreas de abandono proyectado estuvieron localizadas frecuentemente en o alrededor de sitios Natura 2000. Con base en estos resultados, argumentamos que el manejo debería ser fabricado para restaurar los aspectos de la naturaleza que son carentes en cada región. Todavía quedan muchos obstáculos con respecto a la biodiversidad en Europa, pero las especies de megafauna ya se están recuperando. Para potenciar aún más la reintroducción a gran escala, el manejo de Natura 2000 necesitaría incorporar estrategias de reintroducción. Nuestro marco de trabajo puede aplicarse a la evaluación de las oportunidades de reintroducción y a los obstáculos en otras regiones del mundo, y nuestros resultados pueden guiar la redirección de los subsidios para manejar los sistemas socio-ecológicos.

## Introduction

**Figure 1:**
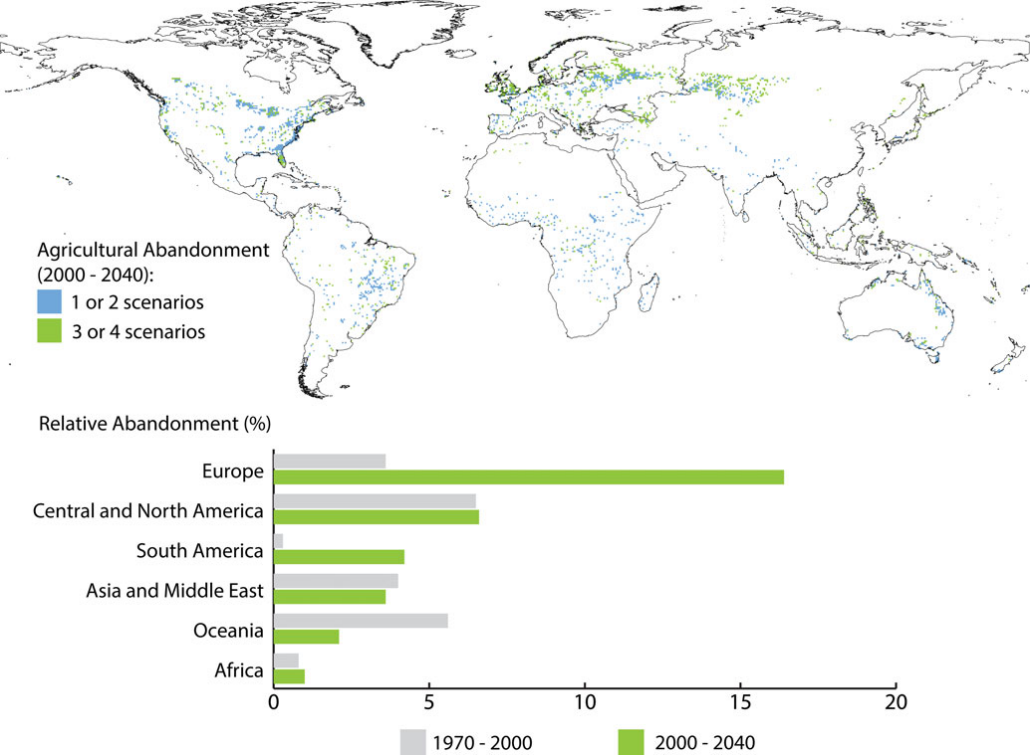
Areas projected to be converted from agriculture to natural areas between 2000 and 2040 based on the IMAGE 2.2 model at a 0.5×0.5 degree resolution (Alcamo et al. 2005) and 4 scenarios of the Millennium Ecosystems Assessment (Alcamo et al. 2005; Cork et al. 2005): Order from Strength (OS), Global Orchestration, TechnoGarden, and Adapting Mosaic. We used the OS scenario for the baseline projections of 2000. The bar graph shows the percentage of past and the projected future conversion from agriculture to natural areas in each world region based on the OS scenario.

Since the development of agriculture, large areas have been converted into farmland across the world (Ramankutty et al. 2002). Changes in technology, productivity, and markets have also led to abandonment of farmland in several instances (Fig. 1). In North America, immigration, population growth, and frontier exploration resulted in the cultivation of huge areas of the continent (Nash 2001). However, due to strong competition from agriculture in the Midwest and the Great Planes, farmland started to be abandoned in the northeastern United States from the middle of the 19th century onward (McGrory Klyza 2001). In tropical regions, many agricultural systems are still based on slash-and-burn techniques, which can be viewed as short-term abandonment (Namgyel et al. 2008; Siebert & Belsky 2014). In Europe, a modeled reconstruction of the land-use changes between 1950 and 2010 suggests that cropland has decreased by almost 19%, whereas pastures and semi-natural grasslands have decreased by almost 6% (Fuchs et al. 2012). Similarly, there has been a decrease in rural population of 17% since the beginning of the 1960s (Navarro & Pereira 2012).

In mountain areas and other marginal lands in Europe, cultivation has provided subsistence to local communities for many years. Upon globalization of agricultural markets and increased labor costs, agriculture in many of these areas is no longer profitable and abandonment occurs (Rey Benayas et al. 2007). However, extensive agriculture has supported high biodiversity of several taxa (Fischer et al. 2012), and there is a strong cultural attachment to these landscapes (Navarro & Pereira 2012). Although there are both species that benefit and species that are negatively affected by farmland abandonment (Sirami et al. 2008; Navarro & Pereira 2012), its impact on biodiversity is often perceived of as solely negative (Queiroz et al. 2014). Much of current European policy and legislation on biodiversity focuses on the protection of habitats and species characteristic of extensive farmland, including through mowing, subsidized grazing, and sowing of grasslands (EC 1979, 1992). Moreover, agri-environmental schemes included in the Common Agricultural Policy of the European Union provide subsidies for the maintenance of traditional agricultural practices (EEA 2004). Despite these policies, farmland abandonment and ecosystem changes are projected to continue in Europe (Verburg & Overmars 2009).

Rewilding has been proposed as an alternative approach to manage farmland abandonment in Europe. There are several approaches to rewilding, from the restoration of Pleistocene ecosystems (Donlan et al. 2006), with an emphasis on reintroduction of extinct species, to the passive management of ecological succession after abandonment, with an emphasis on restoring natural ecosystem processes and reducing the human influence on landscapes (Pereira & Navarro 2015). The latter approach has been called ecological rewilding (Pereira & Navarro 2015).

We developed a framework to explore the opportunities and challenges for ecological rewilding in Europe. We mapped wilderness quality in areas projected to be abandoned by 2040. We define *wilderness* as area of minimum human influence (Carver et al. 2012) as measured here by 4 metrics: artificial light at night (night light) (Sanderson et al. 2002), human accessibility (Carver et al. 2012), proportion of harvested primary productivity (pHPP) (Haberl et al. 2007), and deviation from potential natural vegetation (dPNV) (Rosati et al. 2008). These metrics indicate important human modifications that affect multiple taxa and ecosystem structure (Forman 2003; Haberl et al. 2005; Rich & Longcore 2005; Timmermann et al. 2015). Our hypothesis is that different wilderness metrics lead to the identification of different opportunities and management options for rewilding. We also investigated how current protected area systems support rewilding in and near areas of projected abandonment. We hypothesize that many areas undergoing abandonment are located around Natura 2000 sites, which are often managed for the maintenance of farmland habitats, which poses challenges for rewilding.

## Methods

We used the land-use change projections of the Dyna-CLUE model at a resolution of 1 km^2^ (Verburg & Overmars 2009) to identify areas undergoing farmland abandonment in Europe. Available Dyna-CLUE projections are restricted to the European Union before 2013, the EU27 (27 countries). We used 4 socio-economic VOLANTE scenarios that describe different policy and management choices in Europe (Paterson et al. 2012). We considered abandonment only if it was predicted in at least 3 of the scenarios.

We mapped 4 metrics of wilderness in Europe at a 4 km^2^ resolution. We calculated the pHPP based on the potential net primary productivity and net harvested primary productivity data sets of Haberl et al. (2007). Net harvested primary productivity is the ecosystem productivity appropriated by humans through agriculture or forestry. We mapped accessibility based on travel time considering terrain ruggedness and land-cover data from transport infrastructure to each pixel (Carver & Fritz 1999; EUROSTAT 2006). The dPNV is an estimate of the similarity between the current land cover and the potential natural vegetation (PNV). We used the CORINE 2000 land-cover map for the current vegetation classes (EEA 2012*a*) and the map developed by Bohn et al. (2000) based on expert assessment as the reference PNV. We calculated the night light impact based on high resolution satellite imagery (NOAA National Geophysical Data Center 2012). The light impact score per pixel was the sum of impact scores from the surrounding light sources over a radius of approximately 10 km. These wilderness metrics partially overlapped with parameters used in the Dyna-CLUE model as determinants of land use allocation; therefore, our results should be interpreted carefully. For protected areas, we used the World Database of Protected Areas (World Conservation Union and UNEP-World Conservation Monitoring Centre 2007) and data on the Natura 2000 network (EEA 2012*b*).

We extracted the values of the metrics at the location of projected abandonment from the values calculated at continental level with a bivariate normal kernel function with a radius of approximately 10 km. We split the raster values for all wilderness metrics across the EU27 into quantiles to calculate the amount of farmland abandonment at different ranges of wilderness. We identified the percentage of abandonment areas that fell within the 10%, 25%, 50%, and 75% highest levels of wilderness for accessibility, pHPP, and dPNV. The division into quantiles of the night light data was less precise due to many ties in the values. Therefore, we used the 16.7%, 33.3%, 50%, and 83.3% of the area with the highest levels of wilderness for night light. Because the night light data set was restricted to areas south of 66°N parallel, the quantiles of all metrics were calculated after clipping each data set to this region. We mapped the overlap between dPNV and pHPP by calculating the difference between the normalized values of the 2 metrics. We calculated the projected abandonment around protected areas by measuring Euclidian distance to the borders of protected areas of IUCN category I and II and to Natura 2000 sites. A detailed description of the data sets and methods is in Supporting Information.

These wilderness metrics outline human impacts on biodiversity and ecosystem function. The effects of artificial light are documented for invertebrates (Davies et al. 2012), fish (Becker et al. 2013), birds (Gauthreaux Jr & Belser 2006), and mammals (Boldogh et al. 2007). The strongest effects are direct mortality, modification of community structure, and disruption of migratory routes (Rich & Longcore 2005; Gaston et al. 2013). Furthermore, artificial light produces a night glow effect at distances of several kilometers from the light sources (Kyba et al. 2011). Roads and human accessibility have impacts at individual, species, and community level through direct mortality of several taxa (Forman & Alexander 1998; Forman 2003). Roads and traffic can also cause pollution (Pagotto et al. 2001) and avoidance behaviors in mammals (Whittington et al. 2005; Kitzes & Merenlender 2014), and favor the expansion of invasive species (Vicente et al. 2010) and of human-favored predators (Alterio et al. 1998). The other two metrics, pHPP and dPNV, are indicative of the current ecological and vegetation structures and the amount of primary productivity available within trophic networks. Vegetation type is fundamental in the structuring of ecosystems (Bridgeland et al. 2010), and the amount of primary productivity available in the ecosystems has effects on species abundance (Madhusudan 2004) and richness (Haberl et al. 2005).

## Wilderness metrics in abandonment areas

**Figure 2:**
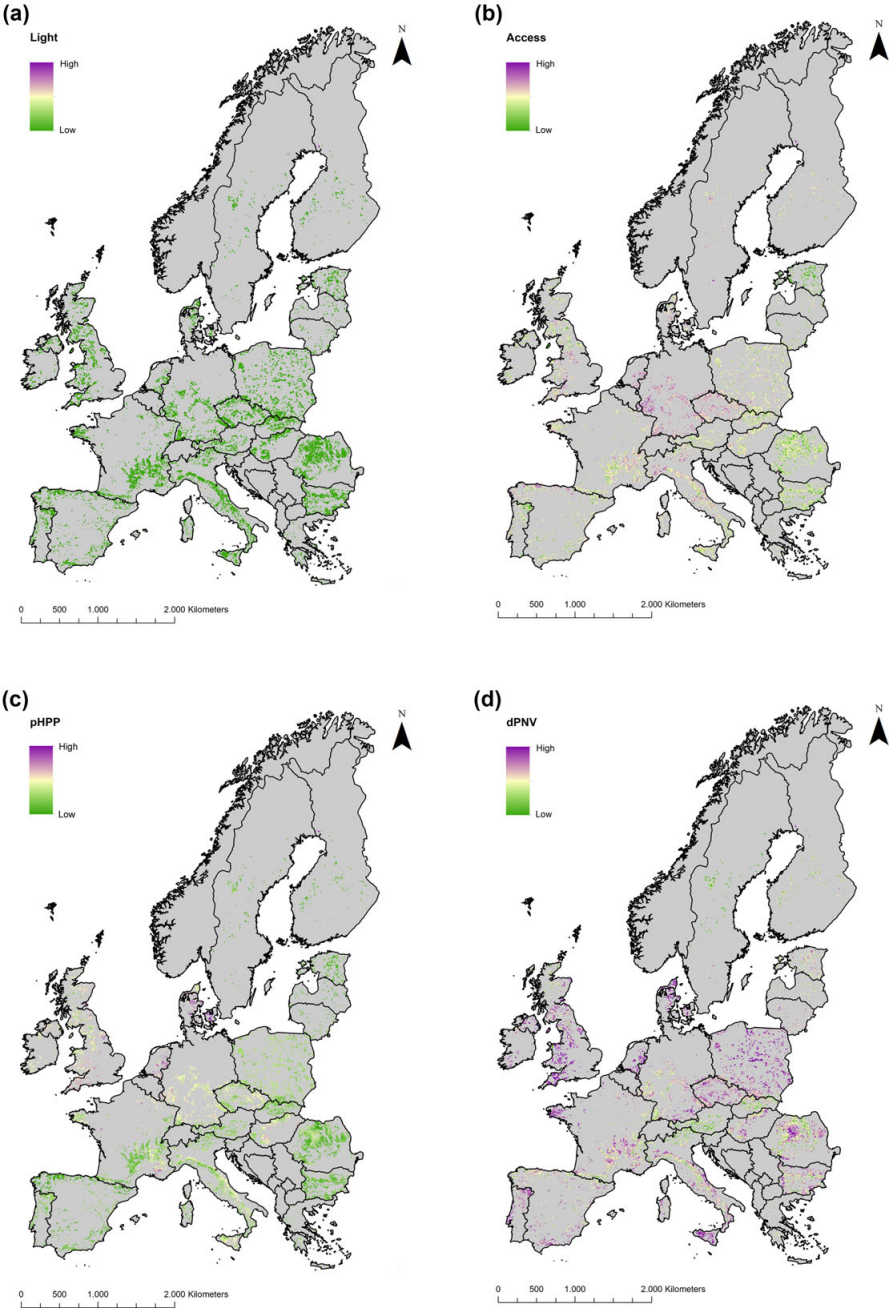
Wilderness value for areas of farmland abandonment based on (a) artificial night light, (b) human accessibility score, (c) proportion of harvested primary productivity, and (d) deviation from potential natural vegetation within a radius of 10 km. High scores of these metrics correspond to low wilderness. The initial resolution of the data sets was 1 km^2^, but pixel size is 3 times larger to increase visibility of the considered areas.

Farmland areas projected to be abandoned in at least 3 scenarios covered 4.2% of the land area in EU27. The maps of wilderness metrics offered snapshots of the current human impact in areas to become abandoned (Fig. 2). More than 87% of abandonment was predicted to occur in the 33% of the area with the highest wilderness as defined by night light (Table1). In contrast, 8.4% of predicted abandonment occurred in the 25% of the area with the highest wilderness as defined by dPNV (Table1). Accessibility and pHPP had intermediate values: 17.4% and 17.5% of abandoned areas were predicted to be, respectively, in the 25% highest wilderness areas as defined by these metrics. This confirms that farmland areas most prone to abandonment exhibit low to moderate levels of infrastructure development and low population density (Navarro & Pereira 2012). Areas of predicted abandonment in central Europe had higher accessibility due to higher infrastructure development than in other parts of Europe (Fig. 2b). Elsewhere on the continent, areas projected to be abandoned are relatively remote rural regions with a long history of landscape modification and low productivity and are often located in mountains, where limits to mechanization make it difficult to compensate for low productivity (MacDonald et al. 2000; Navarro & Pereira 2012).

**Table 1:** Percentage of projected agricultural abandonment within the upper 10%, 25%, 50%, and 75% of highest wilderness values calculated at continental level for the human access score, percentage of harvested primary productivity (pHPP), deviation from potential natural vegetation (dPNV), and night light

Metric	Quantiles of wilderness values			
	10%	25%	50%	75%
Human access	4.4	17.4	47.1	77.7
pHPP	4.7	17.5	48.1	81.7
dPNV	0.6	8.4	43.4	82.1
Night light*	73.7	87.2	91.3	96.8

* For artificial night light, we used 16.7%, 33.3%, 50%, and 83.3% highest wilderness values because the clumping of data does not allow for exact quantile definition.

**Figure 3:**
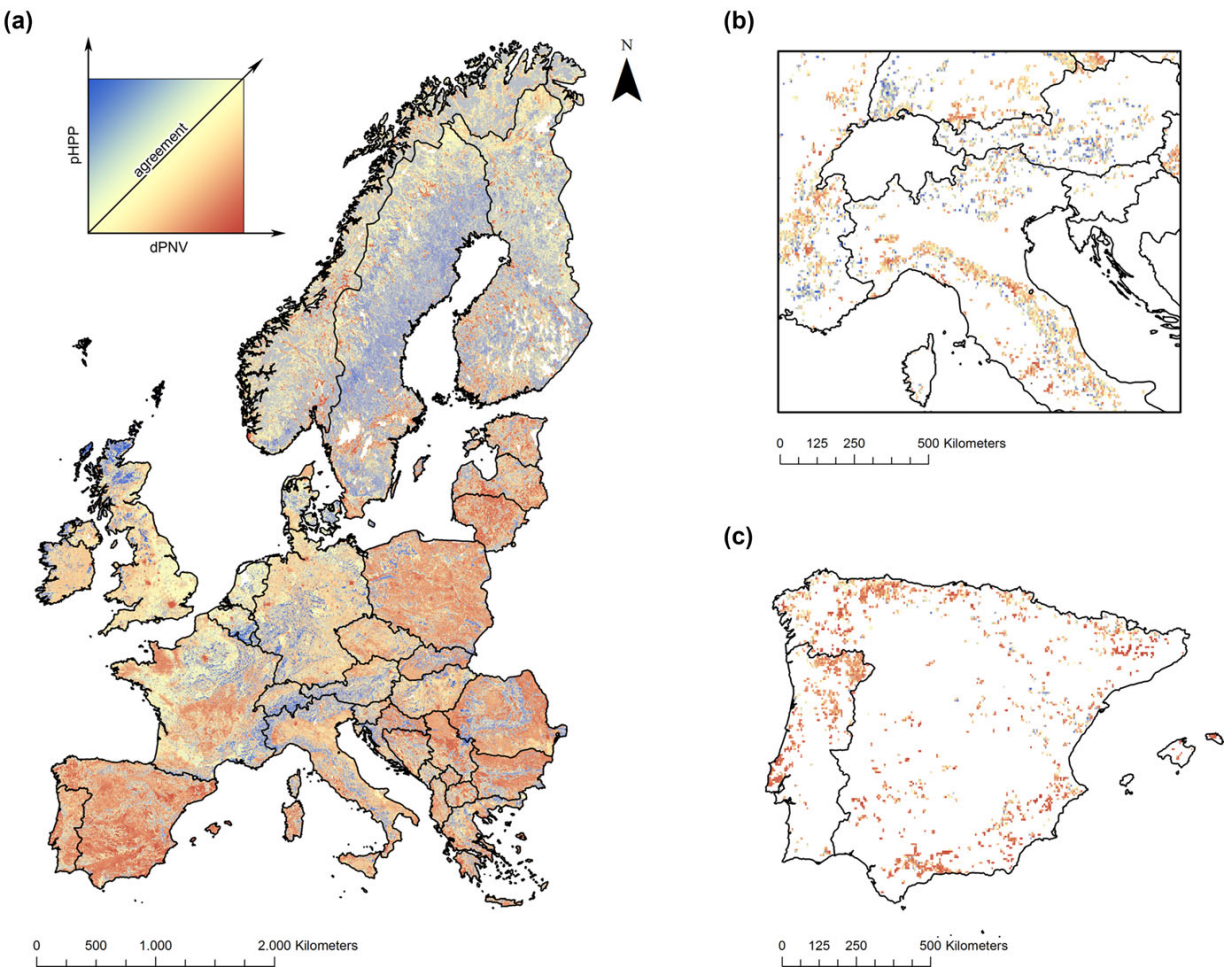
Areas of agreement between the proportion of harvested primary productivity (pHPP) and the deviation from potential natural vegetation (dPNV) in (a) Europe, (b) abandonment locations in the Alps and northern Apennines, and (c) abandonment locations in the Iberian Peninsula (in the online version, yellow represents areas where the normalized values of pHPP and dPNV are equal or close to equal; blue, pHPP is higher than dPNV; red, dPNV is higher than pHPP).The initial resolution of the data sets was 1 km^2^, but pixel size is 3 times larger to increase the visibility of areas considered in (b) and (c).

Identifying areas of agreement and disagreement between pHPP and dPNV at continental and regional scales provides further information on the diversity of local contexts for rewilding (Fig. 3). Although both pHPP and dPNV are strongly related to farming activities, their spatial distribution was quite different (Fig. 3) as a result of underlying environmental drivers, land-use histories, and the degree to which agricultural activities create landscapes closer to or farther away from the natural reference points. Large urban areas such as London, Paris, and Berlin had very high dPNV and very low pHPP (Fig. 3a). In contrast, most mountainous areas showed low dPNV and relatively high pHPP (Fig. 3b), presumably as cattle grazing at high elevations does not produce a high deviation from the original alpine grasslands (Fig. 3b). Areas such as the Iberian Peninsula and large areas of Eastern Europe showed strongly modified vegetation but a lower pHPP than the intensive agricultural regions in Western Europe (Figs. 3a & 3c). This is expected because technological progress has allowed agriculture to gradually intensify in the most productive and easily mechanized lands, whereas climate and biophysical limitations have not allowed some systems, for example in Southern Europe, to increase their productivity above a certain threshold (Pinto-Correia & Mascarenhas 1999). Low levels of mechanization can also be due to economics in areas such as the former socialist countries (Müller et al. 2009) or to local socio-economic factors such as farm size or existing conservation policies. The continuation of low intensity agriculture has nevertheless maintained ecosystems in a modified state throughout many areas of Europe (Ceaușu et al. 2015).

### Protected Areas and Abandonment

In Europe, nationally designated protected areas are based on classifications that often can be mapped to the International Union for Conservation of Nature (IUCN) categories. Protected areas of category I (strict nature reserves and wilderness areas) and II (national parks) directly address the maintenance and support of natural ecological processes and minimum human intervention (Dudley 2008) and therefore would be the most favorable to rewilding. However, protected areas of category I and II occupy only 2.7% of the EU territory (Table2). These areas are biased toward large wilderness areas that have low human presence and thus no agriculture to be abandoned (Dudley 2008; Joppa & Pfaff 2009). As a result, approximately 4% of projected abandonment was inside or within a 5-km radius around protected areas of IUCN category I and II (Table2).

**Table 2:** Proportion of projected agricultural abandonment within a 5-km and a 10-km radius around the protected areas of International Union for Conservation of Nature (IUCN) category I and II and Natura 2000 sites.^a^

		Abandonment	Abandonment in	Abandonment in
Type of protected area	EU27^b^ area	inside	a 5 km radius	a 5–10 km radius
IUCN category I and II	2.7	1.2	2.9	3.6
NATURA 2000	17.9	14.4	31.9	22.1

a The areas of the radii are not overlapping and do not contain the areas inside the protected areas.

b European Union before 2013 (27 countries).

National systems of protected areas coexist with Natura 2000, the European Union system of protected areas. The Natura 2000 network occupies almost 18% of the EU territory (Table2) and aims to maintain specific species and habitats in a “favorable conservation status” (EC 1979, 1992). Many of the species and habitats under the Natura 2000 management guidelines are characteristic of extensive farmland and early successional habitats (Halada et al. 2011; Prach et al. 2013). Almost half of projected abandonment was predicted to occur in or within a 5-km radius of Natura 2000 sites (Table2). Therefore, to potentiate rewilding in those regions, Natura 2000 management guidelines have to be expanded to include rewilding actions.

### Policies and Management for Rewilding

**Figure 4:**
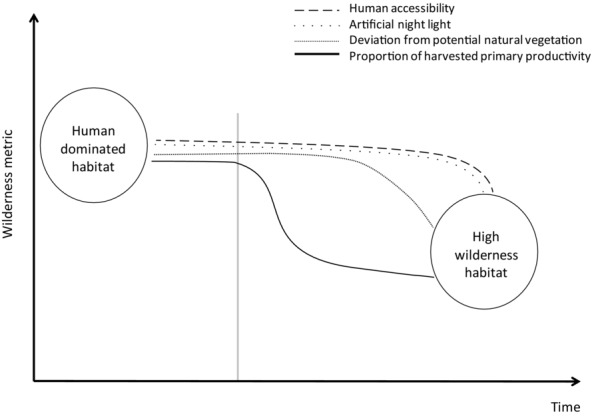
Conceptual illustration of the variation over time in wilderness value after abandonment based on 4 metrics (gray vertical line, beginning of farmland abandonment). High values of each metric correspond to low wilderness.

The speed at which different dimensions of wilderness will respond to farmland abandonment varies. The pHPP will respond almost immediately (Fig. 4) because land abandonment, even if progressive or partial, corresponds to a decrease in the appropriation of ecosystem productivity. Decreased pHPP can lead to the restoration of natural vegetation and a decrease in dPNV. However, several obstacles make it difficult not only to predict the amount of time taken by ecosystems to reach a new equilibrium but also to predict how close the novel ecosystems will be to the PNV (Vera 2000; Rey Benayas et al. 2007) (Fig. 4). Climate change may lead to modified patterns of PNV (Hickler et al. 2012). Additionally, levels of natural herbivory and other disturbances to natural succession (e.g., fire, flood, wind) will be distinct in post-abandonment landscapes in present Europe from the Pleistocene or pre-agricultural Holocene (Fuhlendorf et al. 2009). Thus, management actions to increase populations of wild herbivores through no-hunting zones or reintroductions could promote the restoration of natural vegetation. Moreover, the recovery of forest vegetation is often hindered by the isolation of current seed banks (Rey Benayas et al. 2008). In some areas, local forest species have been replaced by non-native species planted mainly for commercial purposes, and the structure and composition of these communities differ from those of native communities (Proença et al. 2010). Planting of woodland islets with native trees could accelerate rewilding (Rey Benayas & Bullock 2015).

Other dimensions of wilderness may also have a delayed response to abandonment. Artificial light may decrease soon after abandonment, but due to the presence of public light infrastructure and the development of new activities in the landscape, such as tourism (Cerqueira et al. 2015), some degree of artificial light may persist for long periods. Policies can promote the progressive decrease of public lighting and foster tourism infrastructure that uses low light pollution architecture (Salmon 2006). Accessibility may be the slowest to respond to abandonment because roads will persist for a long time. Still, a decrease in traffic could lead to a decrease in the effects of road mortality on animal populations (Forman 2003) and a decrease in other negative effects such as noise and pollution (Summers et al. 2011). Policies could promote decreased accessibility by promoting road removal or implementing traffic limitations (Switalski et al. 2004).

### Biodiversity Dynamics of Rewilding

Rewilding will often result in the increase of forest cover, leading to many specialist species of open areas becoming less abundant and more spatially restricted. Common farmland birds and grassland butterflies are already becoming less abundant (Tryjanowski et al. 2011; Van Swaay et al. 2012), although much of this decrease is probably attributable to agriculture intensification (Donald et al. 2006). At the same time, several species in Europe are taking advantage of the spaces and resources made available by land abandonment, such as the gray wolves (*Canis lupus*) and brown bears (*Ursus arctos*) (Enserink & Vogel 2006; Gehrig-Fasel et al. 2007; Chapron et al. 2014). This megafauna increase is also an outcome of decades of conservation policies (e.g., Hoffmann et al. 2010; Deinet et al. 2013; Navarro & Pereira 2015), including species protection regulations such as the Habitats and Birds Directives and national legislations; the implementation of national protected areas and the Natura 2000 network; and reintroduction programs of keystone and emblematic species, such as the European bison (*Bison bonasus*) (Kuemmerle et al. 2010) and the Iberian lynx (*Lynx pardinus*).

Current populations of megafauna spatially coincide with high values of wilderness metrics and with projected areas of abandonment, especially in mountainous areas, thus raising the possibility of migration into the newly available space (Ceaușu et al. 2015). Moreover, rewilding will increase connectivity of natural habitats, supporting the adjustment of ranges to climate change (Lindner et al. 2010).

How biodiversity dynamics will continue to evolve after abandonment and what rewilding strategies should be implemented are active areas of research. Timmermann et al. (2015) showed that despite management interventions to maintain extensive farmland in Denmark, vegetation structure continued to change. Some scientists argue that pre-farming levels of herbivory were sufficiently high to maintain a mosaic of woods and grasslands (Vera 2000; Sandom et al. 2014). Thus, several approaches to rewilding in Europe are based on filling the ecological role of extinct wild herbivores (Vera 2000; Monbiot 2013). However, several recent studies suggest that Europe was mostly covered by closed canopy forests until humans created open landscapes (Birks 2005; Mitchell 2005). We hypothesize that in former and novel landscapes, fire, storms, and diseases could generate a fluid mosaic of early successional habitats in a predominantly closed forest (Navarro et al. 2015). An open question is whether large herbivores can delay succession by selectively grazing open areas, particularly in the presence of predators. In any case, one would not expect a lack of open habitats in a post-abandonment Europe, including remaining agricultural areas and areas where abiotic factors limit tree recruitment, such as high elevation areas and wetlands.

### A Global Perspective on Abandonment and Rewilding

Reponses to farmland abandonment differ across the world. In several regions, such as Australia, there are few agricultural subsidies (Productivity Commission 2005), and abandonment has been taken up as an opportunity for restoration of native vegetation (Cramer et al. 2007). In other countries, agricultural subsidies have been implemented to halt abandonment. Many of these subsidies are justified by environmental concerns but are also driven by socio-economic considerations (Mattison & Norris 2005; Batie 2009).

Agricultural policies have also changed over time, subject to globalization trends and protectionist tendencies (Mattison & Norris 2005). In the 19th century, the response to abandonment in the northeastern United States was the acquisition of land by government to encourage reforestation and restoration (McGrory Klyza 2001). During the economic depression of the 1930s, agricultural subsidies were designed as a support for farmers. In the more recent decades, they have also addressed environmental issues (Mattison & Norris 2005). In the past, the emphasis of these measures was to provide incentives for setting aside areas for wildlife habitat (Haufler et al. 2005). But funding has now shifted toward mitigating the impacts of agricultural intensification and the funding for wildlife habitat has decreased (Mayrand et al. 2003). Many previously set aside areas have now been brought back into production, especially for biofuels (Avery 2006).

Wilderness mapping can support the development of rewilding strategies in these different agricultural contexts. Our analyses confirmed our hypotheses that different wilderness metrics reveal different priorities and that abandonment areas in Europe are close to Natura 2000 sites. Rewilding actions can be prioritized toward improving the wilderness metrics lacking in a certain region (e.g., decreasing infrastructure in areas of high accessibility). The management of protected areas can also be used to facilitate rewilding in areas of high abandonment. In marginal agricultural regions where agricultural subsidies are politically difficult to remove, subsidies can be shifted to rewilding measures such as the creation of no-hunting zones and wildlife habitat (Merckx & Pereira 2014).

Conservation management in the face of anthropogenic change represents an issue of global importance. Soulé (1985) argues that the role of conservation should be to protect nature for its intrinsic value and ensure protection for the least disturbed ecosystems. Kareiva and Marvier suggest instead that conservation should focus on human modified systems because ecological dynamics are tightly connected to human dynamics (Kareiva & Marvier 2012). A rewilding approach recognizes that the majority of ecosystems have been modified by humans, but identifies opportunities for decreasing the human pressure on ecosystems and restoring the more natural biodiversity dynamics and ecosystem services associated with wilderness (Naidoo et al. 2008; Cerqueira et al. 2015).

## Supporting Information

A detailed description of the data sets and methods (Appendix S1) are available on-line. The authors are solely responsible for the content and functionality of these materials. Queries (other than absence of the material) should be directed to the corresponding author.
