# Supplementary material for: Mapping opportunities and challenges for rewilding in Europe
Source: Conserv Biol. 2015 May 21;29(4):1017–27. doi: 10.1111/cobi.12533 (PMC4584510; doi:10.1111/cobi.12533)
Supplement: Supplementary file 1 — A detailed description of the data sets and methods (Appendix S1) are available on-line. The authors are solely responsible for the content and functionality of these materials. Queries (other than absence of the material) should be directed to the corresponding author. [file cobi0029-1017-sd1.docx]

**Supplementary information**

**Description of datasets**

**Projections of farmland abandonment – Global scale**

We used for Figure 1 global land-cover maps produced for the Millenium Ecosystems Assessment (MA) for 1970, 2000, and 2040 based on the IMAGE 2.2 model at a 0.5 by 0.5 degree resolution (Alcamo et al. 2005). We chose the Order from Strength maps as a baseline for 1970 and 2000, and used the projections with the four scenarios of the MA for 2040: Order from Strength, Global Orchestration, Technogarden, and Adapting Mosaic (Cork et al. 2005). For each scenario, we identified cells that were classified as agriculture in the baseline map of 2000, and were classified as natural in 2040. Thus, those cells represent the "agricultural abandonment and revegetation". Combining all four maps provides a global map of abandonment and revegetation indicating the level of agreement between scenarios, i.e. whether a cell was considered as abandoned and restored in one and two scenarios, or in three or four scenarios (see legend of Figure 1).

We calculated, per continent, the ratio between the number of cells classified as abandoned and restored in at least 3 scenarios in 2040, and the number of cells classified as agriculture in the 2000 baseline (see bar plot, white bars). The same ratio was calculated for the two baseline maps of 1970 and 2000 (see bar plot, black bars). Note that the projections do not distinguish between natural and planted forest.

**Projections of farmland abandonment – European scale**

We use the farmland abandonment projections of the Dyna-CLUE model (Verburg & Overmars 2009) based on four socio-economic scenarios (Paterson et al. 2012). Similarly to the well-known Special Report on Emissions scenarios of the Intergovernmental Panel on Climate Change (IPCC 2013) these scenarios range across two axes: regionalization versus globalization; and inclination versus aversion towards sustainable lifestyle choices and the level of regulation to achieve these. As an indicator of areas with a relatively high chance of facing abandonment, we chose for our analysis the areas of farmland abandonment predicted in at least three of the four scenarios.

The farmland abandonment projections of the Dyna-CLUE model (Verburg & Overmars 2009) have a resolution of 1 km^2^. This model has a better resolution than the model used for Figure 1 and it was constructed specifically for the European context, thus we preferred to use it for our analyses. This model combines a land-use allocation module based on land demand at regional level, with a bottom-up module describing land-use conversions determined by local processes.

**Wilderness metrics datasets**

We lowered the datasets of all the wilderness metrics to the lowest resolution available among the wilderness metrics datasets. In our analysis, all metrics had a resolution of 4 km^2^.

We calculate proportion of harvested primary productivity (pHPP) based on the datasets provided by Haberl et al. (2007) for net potential primary productivity (PP_0_) and net harvested primary productivity (HPP). We calculated pHPP as the ratio between HPP and PP_0_. Haberl et al. (2007) have derived potential primary productivity (PP_0_) from the Lund-Potsdam-Jena dynamic global vegetation model (Sitch et al. 2003) and harvested primary productivity (HPP) based on the data of the Food and Agriculture Organization (FAO). The FAO data is based on national statistics which can have different degrees of accuracy (Haberl et al. 2007) and thus it presents some limitations.

For the deviation from potential natural vegetation (dPNV) dataset, we used the potential natural vegetation (PNV) map developed by Bohn et al. (2000) based on expert assessments. We used the CORINE 2000 land cover classes to compare PNV with current land cover and calculate the deviation from each other. In order to estimate the probability of coincidence, we classified the relationships between the current land classes and the PNV classes according to four different scores: 1 = assumed coincidence, 0.75 = most probable, 0.5 = probable and 0.1 = possible. To correct for anthropogenic pressure in terms of livestock grazing impacts on semi-natural grassland, we also integrated FAO data on grazing density that was linear transformed to the interval [0-1], with 1 representing a density of 20 heads/km² or more (FAO 2006). There are some criticisms regarding the PNV dataset. The more controversial regions are areas of the Iberian peninsula and of the Pannonian region (Hickler et al. 2012). These are areas in which our PNV data indicate forests as the natural vegetation but the dry climate in southern Europe, for instance, could also favor a shrubland type of vegetation (Hickler et al. 2012).

For the accessibility data, several datasets were aggregated: travel time as a measure of remoteness, night lights as a measure of the absence of artificial structures and population density (EUROSTAT 2006). The last two datasets were scaled directly to the [0-1] interval while. The travel time was calculated using a cost-distance approach based on the Naismith’s Rule of different relative travelling times (Carver & Fritz 1999) based on the data from the Eurogeographics Roads and Open Street Map databases. The results were adjusted for terrain ruggedness based on data from the Shuttle Radar Topography Mission at 1 km resolution (SRTM) and for land cover based on CORINE 2000 and 2006. The adjustment of traveling times was done through a GIS-grid-based model in which steep slopes (>40^o^) have a negative impact on traveling time by forcing a traveller to circumvent them. The Naismith’s Rule assumes that a person can walk at a speed of 5km/h on flat terrain and with decreasing speed for ascending and descending slopes, depending on the degree of the slope. Additionally, a relative cost surface is assumed for all land-cover types where, for instance, marshland and forests require longer travel times than grasslands and pastures. Heather and forest are assumed to have walking times of 3km/h while bogs would be crosses at 2 km/h. Rivers and water bodies were considered as absolute barriers, with the exception of the cases where a bridge exists. The travel time, night light and population density datasets were then combined with equal weights for the final human accessibility layer.

We mapped the impact of artificial night light within a spectral range of 0.5 – 0.9 µm by applying a normal kernel function over a radius of approximately 10 kilometers to account for both ecological and sensorial effects regarding the human perception of wilderness (Longcore & Rich 2004; Kyba et al. 2011). The data were obtained from the Visible Infrared Imaging Radiometer Suite (VIIRS) of the Suomi National Polar-orbiting Partnership (SNPP) for the year 2012 (NOAA National Geophysical Data Center 2012) with a spatial resolution of 15 arc seconds. Artificial night light data for the European areas north of approximately 66^o^ latitude is missing from the original dataset. We therefore excluded these areas also from the other wilderness metrics datasets when doing comparisons between these data.

**Analyses**

**Extraction of wilderness values at the locations of potential abandonment and rewilding**

All data extraction and analyses were performed in ArcGIS v10.2.1 (Esri, California, USA). For the extraction of metrics values at abandonment locations, we used a bivariate normal kernel function that covers a circular area of a radius of approximately 10 km in order to account for the surrounding areas but give increasing importance to points closer to the locations of abandonment . We chose a radius of 10 km for our wilderness calculation in order to approximate the maximum typical distance for seed dispersal, taking into account the contribution of both biological and physical dispersers (Clark et al. 1999; Nathan & Muller-Landau 2000), from possible source populations into the newly abandoned areas (Rey Benayas et al. 2008). Such distances are also a good approximation for the dispersal of other species important for wild ecosystems such as carnivores and large herbivores (Sutherland et al. 2000). The weighting of the surrounding areas according to the distance from abandonment is due to the fact that closer areas will have a stronger effect in terms of species dispersal.

**Combination of the functional wilderness metrics**

We combined pHPP and dPNV by normalizing the values for both metrics for the [0, 1] interval according to the formula: $x_{n}= \frac{x-x_{min}}{x_{max}-x_{min}}$where *x_n_* is the normalized value, *x* is the initial value of the wilderness metrics, and *x_min_* and *x_max_* are the minimum and the maximum values for either of the metrics. We then performed the difference dPNV – pHPP. The results of this operation were then symbolized in Figure 2, with positive numbers (higher normalized dPNV) represented by the lower color ramp and the negative numbers (higher normalized pHPP) represented by the upper color ramp of the legend.

**Calculation of abandonment levels in wilderness quantiles**

In order to calculate the amount of farmland abandonment at different ranges of wilderness, we divided the overall raster values for all wilderness metrics at continental scale into quantiles. We identified the amount of abandonment points that falls within 10%, 25%, 50% and 75% highest wilderness levels for accessibility, pHPP, and dPNV. Due to the clustering of the night light data, the division into quantiles was less precise. Therefore, we used the highest 16.67%, 33.33%, 50% and 83.33% of the area for artificial night light.

**Calculation of abandonment levels in protected areas**

We calculated the Euclidean distance from places of farmland abandonment to the borders of protected areas of IUCN category I and II and to Natura 2000 sites (Table 2). Euclidian distance is a useful simplification for connectedness in our context but it is worth pointing out that from the point of view of ecological processes, a short Euclidian distance does not guarantee opportunities for dispersal and migration for taxa with different spatial requirements (Goldberg & Lande 2007).

**Bibliography**

Bohn, U., G. Gollub, C. Hettwer, Z. Neuhäuslová, T. Raus, H. Schlüter, and H. Weber. 2000. Karte der natürlichen Vegetation Europas, Maßstab 1: 2 500 000.[Map of the Natural vegetation of Europe. Scale 1: 2 500 000]. Bundesamt für Naturschutz, Bonn.

Carver, S., and S. Fritz. 1999. Mapping remote areas using GIS. Landscape character: Perspectives on management and change. Natural Heritage of Scotland Series, HMSO:112–126.

Clark, J. S., M. Silman, R. Kern, E. Macklin, and J. HilleRisLambers. 1999. Seed dispersal near and far: patterns across temperate and tropical forests. Ecology **80**:1475–1494.

EUROSTAT. 2006. GEOSTAT population grid 2006.

FAO. 2006. Health Atlas (GLIPHA). Global livestock production and health atlas of the FAO. Food and Agriculture Organization of the United Nations, Rome, Italy.

Goldberg, E. E., and R. Lande. 2007. Species’ Borders and Dispersal Barriers. The American Naturalist **170**:297–304.

Haberl, H., K. H. Erb, F. Krausmann, V. Gaube, A. Bondeau, C. Plutzar, S. Gingrich, W. Lucht, and M. Fischer-Kowalski. 2007. Quantifying and mapping the human appropriation of net primary production in earth’s terrestrial ecosystems. Proceedings of the National Academy of Sciences **104**:12942.

Hickler, T. et al. 2012. Projecting the future distribution of European potential natural vegetation zones with a generalized, tree species-based dynamic vegetation model. Global Ecology and Biogeography **21**:50–63.

IPCC. 2013. Climate Change 2013: The Physical Science Basis. Contribution of Working Group I to the Fifth Assessment Report of the Intergovernmental Panel on Climate Change [Stocker, T.F., D. Qin, G.-K. Plattner, M. Tignor, S.K. Allen, J. Boschung, A. Nauels, Y. Xia, V. Bex and P.M. Midgley (eds.)]. Page 1535. Intergovernmental Panel on Climate Change, Cambridge University Press, Cambridge, United Kingdom and New York, NY, USA.

Kyba, C. C., T. Ruhtz, J. Fischer, and F. Hölker. 2011. Cloud coverage acts as an amplifier for ecological light pollution in urban ecosystems. PLoS One **6**:e17307.

Longcore, T., and C. Rich. 2004. Ecological light pollution. Frontiers in Ecology and the Environment **2**:191–198.

Nathan, R., and H. C. Muller-Landau. 2000. Spatial patterns of seed dispersal, their determinants and consequences for recruitment. Trends in ecology & evolution **15**:278–285.

NOAA National Geophysical Data Center, E. O. G. 2012. VIIRS Nighttime Lights - 2012. NOAA National Geophysical Data Center.

Paterson, J., M. Metzger, and A. Walz. 2012. Deliverable No: 9.1 - The VOLANTE scenarios: framework, storyline and drivers. VOLANTE - Visions of land use transitions in Europe.

Rey Benayas, J. M., J. M. Bullock, and A. C. Newton. 2008. Creating woodland islets to reconcile ecological restoration, conservation, and agricultural land use. Frontiers in Ecology and the Environment **6**:329–336.

Sitch, S., B. Smith, I. C. Prentice, A. Arneth, A. Bondeau, W. Cramer, J. O. Kaplan, S. Levis, W. Lucht, and M. T. Sykes. 2003. Evaluation of ecosystem dynamics, plant geography and terrestrial carbon cycling in the LPJ dynamic global vegetation model. Global Change Biology **9**:161–185.

Sutherland, G. D., A. S. Harestad, K. Price, and K. P. Lertzman. 2000. Scaling of natal dispersal distances in terrestrial birds and mammals. Conservation Ecology **4**:16.

Verburg, P. H., and K. P. Overmars. 2009. Combining top-down and bottom-up dynamics in land use modeling: exploring the future of abandoned farmlands in Europe with the Dyna-CLUE model. Landscape Ecology **24**:1167–1181.
